# Making Space for Permanent Molars in Growing Baboon (*Papio anubis*) and Great Ape (*Pan paniscus* and *P. troglodytes*) Mandibles: Possible Ontogenetic Strategies and Solutions

**DOI:** 10.1155/2011/484607

**Published:** 2011-06-07

**Authors:** Julia C. Boughner

**Affiliations:** Department of Anatomy and Cell Biology, University of Saskatchewan, Health Sciences Building B328, 107 Wiggins Road, Saskatoon, SK, Canada S7K 5E5

## Abstract

While mandible proportions do not appear to constrain permanent molar initiation times, how adequate space is created in the corpus for these teeth in a timely way is not well understood. This question is important for explaining how primate tooth and jaw development and evolution are coordinated. Landmark and linear measurement data were used to characterize mandible shape, growth trajectory, and growth rate between two genera, *Papio* and *Pan*, with contrasting permanent molar initiation schedules and mandible proportions. 3D geometric morphometric and 2D bivariate analyses showed genus-level differences in mandible morphology from birth that were amplified by different postnatal growth trajectories. Different corpus proportions and regional variation in corpus growth rates helped create space in a timely way for the molars. Regional corpus growth rates may evolve alongside permanent molar morphology and developmental timing to modify space available in the corpus for these teeth.

## 1. Introduction

Primate mandible morphologies and times of permanent molar initiation, used here to define the start of odontogenesis, vary widely across taxa. How sufficient space is created for the developing permanent molars in a growing mandible of a particular morphology at appropriate times is not well understood. Once emerged, the deciduous dentition maintains a large proportion of the space in the mandible corpus required for the permanent antemolar teeth. But for the permanent molars, space must be created anew via the growth of the jawbone. The timing of permanent molar initiation as well as molar mineralization rates and periods varies, often markedly, among primates [1–8]. Times of permanent molar initiation are not likely to be constrained by a lack of space for these teeth in the growing jaw [9]. Other work also suggests ontogenetic if not evolutionary autonomy between the teeth and the mandible [10–17], where teeth experience stronger selection pressures than do the jaws and face [17–19]. At least in African apes, the growth of tooth-bearing regions of the mandible is less plastic than that of edentulous regions of the jaw, notably areas of muscle attachment [20]. There is also evidence based on African ape data of developmental decoupling among various regions of the mandible corpus, ramus, condyle, and alveolar versus cortical bone that appears to be a response to the different functions of these skeletal tissues [20]. Specifically, the presence and development of the dentition may influence mandible corpus form and growth [21–24]. For example, the timing of permanent tooth initiation, emergence, and eruption may drive rates of corpus growth [21]. Regardless of whether or not the teeth directly impact mandible growth, how space is made in the mandible in a timely way for the permanent molars of species with different jaw proportions and tooth initiation schedules is a problem that is not well understood and warrants study.

The current work builds on a previous geometric morphometric test of whether space in the mandible constrained times of permanent molar initiation using *Papio anubis*, *Pan troglodytes*, and *Pan paniscus* as models [9]. Using 3D molar crypt and crown landmark and measurement data, this study found no significant difference in the space available for successive permanent molars among these three species, suggesting that mandible proportions do not constrain molar initiation times [9]. How the olive baboon and African ape mandibles grow to accommodate, respectively, more or less staggered times of permanent molar initiation needs investigating to better understand the mechanisms that coordinate primate tooth and jaw growth and evolution.

Using mandible 3D landmark data alongside 2D mandible linear measurement data, this study examines the ontogenetic changes in the proximal part of the corpus in which the permanent molar teeth form. Average mandible length is greater in adult baboons (*Papio anubis*) compared to adult chimpanzees (*Pan troglodytes*) and bonobos (*Pan paniscus*). Relative to the apes, baboons have not only more prognathic jaws but also anteroposteriorly longer permanent molar crowns. The initiation of successive permanent molars is more staggered over time in *Papio* than in *Pan*. However, total duration of permanent molar crown mineralization and, indeed, overall somatic maturation is shorter in *Papio* compared to *Pan* by at least three years [25, 26]. Hence, relative to *Pan*, mandible growth in *Papio* is probably accelerated in order to achieve adult proportions that accommodate earlier forming and, in some cases, anteroposteriorly longer permanent molars. First, this study uses geometric morphometrics to compare mandible shapes and growth trajectories between *Papio* and *Pan*. Second, this work contrasts linear dimensions and growth rates of *Pan* and *Papio* mandible corpora in order to characterize how space is created in the jawbone for the permanent molars.

## 2. Materials

*Papio* and *Pan* are apt models for this study because they have contrasting mandible shapes, permanent molar initiation times, and molar mineralization periods. In the longer baboon mandible, the initiation times of successive permanent molars are more staggered; each molar crown is almost completely mineralized before the next molar begins to develop. In the shorter ape mandibles, the initiation of successive permanent molars is more rapid; molars begin to form in quicker succession with greater overlap in time. Thus *Papio* would appear to be a good model of a molar initiation pattern for a jaw with little to no “extra” space in it. Conversely, with more than one molar crown mineralizing at a time, *Pan* would appear to be a good model of a molar initiation pattern for a jaw with “extra” space in it. Ontogenetic change in mandible shape and size was studied in 52 baboons (*Papio anubis*), 59 chimpanzees (*Pan troglodytes*), and 44 bonobos (*Pan paniscus*). The olive baboon skulls were housed in the Natural History Museum (NHM) and the Royal College of Surgeons (RCS), both in London, UK. The chimpanzee material was housed at the NHM and the Powell-Cotton Museum (PCM), Kent, UK. The bonobo collection was housed in the Royal Museum of Central Africa (RMCA), Tervuren, Belgium. All three species were sampled across as broad and as comprehensive range of developmental ages as possible while balancing the numbers of each sex per species (Table 1). 

Four age groups were defined (Table 2) within this developmental continuum, the ranges of which are explained in the next section. Briefly, age group included individuals of known (male, M; female, F) and unknown (U) sex: *Papio anubis*, *M* = 25, *F* = 23, and *U* = 3; *Pan paniscus*, *M* = 22, *F* = 17, and *U* = 5; *P. troglodytes*,  *M* = 25, *F* = 22, and *U* = 12. These wild-shot animals were sexed using teeth, external genitalia, and nipples, and in a few cases, other external evidence [27, 28]. All specimens were pathology-free.

## 3. Methods

Bonobo (*Pan paniscus, n* = 44), chimpanzee (*Pan troglodytes, n* = 60), and olive baboon (*Papio anubis, n* = 60) mandibles were radiographed and assigned an approximate relative dental age (ARDA) from their dental development (detailed in [9, 29]). Age ranges corresponded to equivalent stages of ontogenetic growth and sexual maturity between *Papio* and *Pan*. Specimens were then assigned to one of four age groups: infants, younger and older juveniles, and adults based on their ARDA (Table 2).

**Figure 1:**
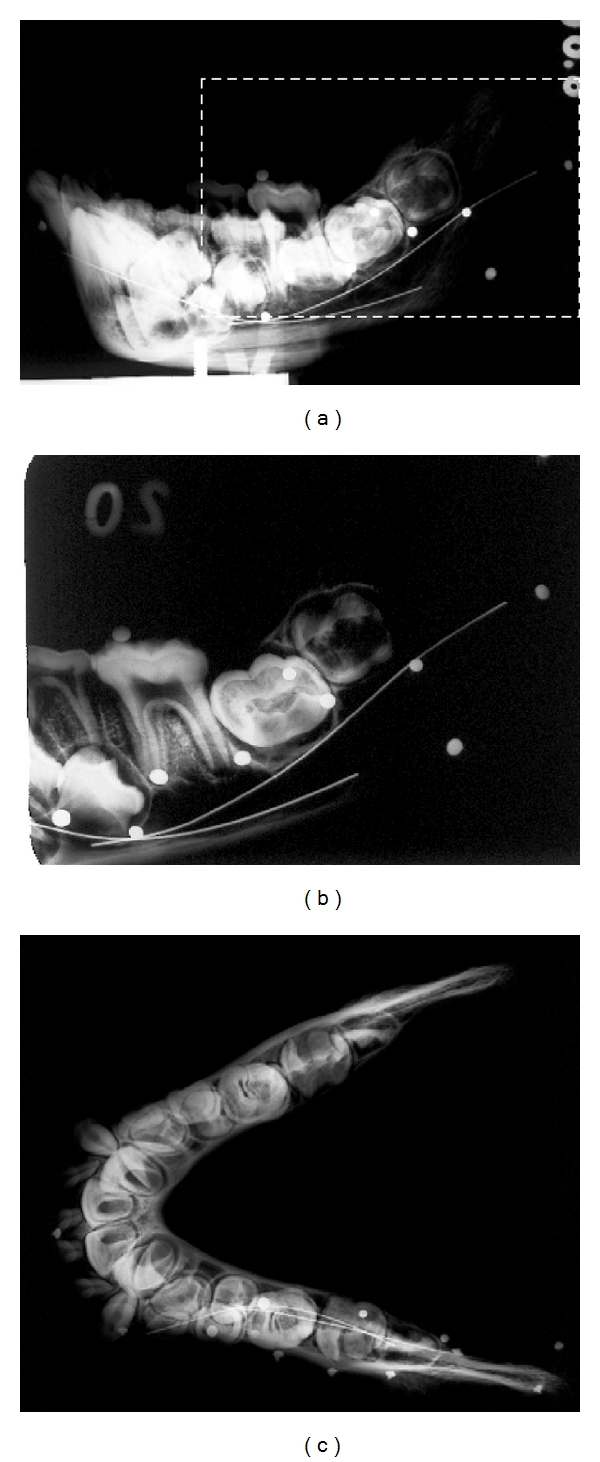
Example of radiographic image data used to determine approximate relative dental age (ARDA): *Pan troglodytes* mandible in lateral (a), intraoral (b) and dashed box in panel (a), and occlusal views.

Both the NHM and RCS specimens were radiographed with a Phillips Industrial Unit. A portable Faxitron Radiographic System (model 8040-310, Field Emission Limited) was used to radiograph specimens from the RCS and RMCA. Lateral and occlusal views of each mandible (Figures 1(a) and 1(c)) were taken with Kodak Industrex X-Ray Film AA400-5. Lingual intraoral views of the molars (Figure 1(b)) were taken with Kodak Ultra-Speed Dental Film DF-50 Size 4.

**Figure 2:**
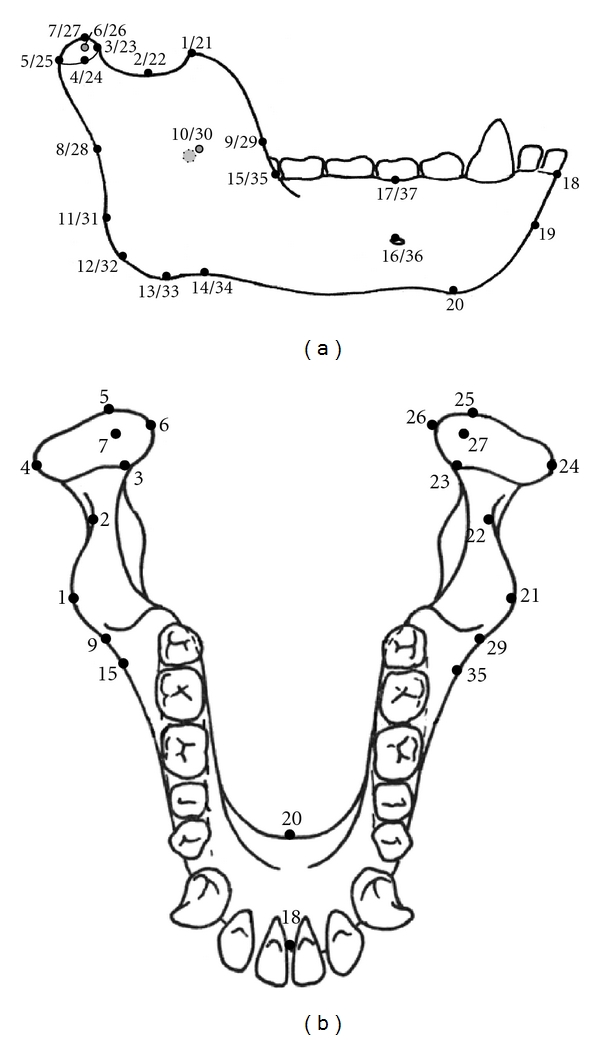
Mandible landmarks: (a) buccal view and (b) occlusal view.

Surface landmark data were collected from each mandible using a MicroScribe 3DX (Immersion Corp). All raw landmark coordinates were logged directly into a Microsoft Excel spreadsheet via InScribe software (Immersion Corp). Thirty-seven 3D mandible landmarks were created on the strengths of their homologies between species [30–32] and by how appropriately they visually represented overall mandible morphology and ontogenetic change in morphology (Table 3, Figure 2). Landmark data were analyzed using software program *morphologika* [33]. Differences of size, translation, and rotation were eliminated using Generalised Procrustes Analysis (GPA) [34–36] before executing a Principal Components Analysis (PCA) on the data to describe the principal vectors of shape variance between *Papio* and *Pan*. A GPA was chosen for the ability to visualize ontogenetic shape change and analyze variance using PCA, particularly as mandible and molar landmarks were created to describe morphological change among taxa and across growth. Shape variance was rendered visually in 3D, first using only landmarks, and second using a constructed “wireframe” that linked adjacent landmarks according to user specifications. These vectors, or principal components (PCs), were used to explore the relationship between shape and other factors such as centroid size, the square root of the sum of squared distances of a set of landmarks from their centroid. Calculated during GPA, centroid size is equally uncorrelated with all landmarks in a given data set and thus is an unbiased measure of size for an object defined by multiple 3D landmarks. 

Principal components analyses were run on a single set of all ape and baboon landmark data to compare mandible development, and on separate sets of landmark data for each species. Permutation tests determined if mandible ontogenetic trajectories were statistically different between genera. Using the PC scores from a PCA, the software program Perm PCA [37] reiteratively tested the statistical significance of the angle between species vectors of shape change across a given PC (significance = 0.05). For this test, a PCA of the GPA data was run for all selected landmarks for two groups. The angle of Group 1 and then of Group 2 to the selected PC were extracted. The angle between these two angles was then computed. Next, individuals were randomly reassigned to either Group 1 or Group 2, with the same number of individuals assigned to each new group as the original groups. Finally, the angle between these groups was recomputed. All this was done for one thousand iterations. The number of times that the permuted angles were smaller than the initially computed true angle yielded the *P* value. 

To determine if mandible shape differed significantly between species throughout ontogeny, the mean shape of the GPA-registered landmark data was calculated for each of the four age groups in both genera and run through permutations tests of difference of means (*P* = .5). This was a direct and assumption-free test of the significance of differences between infant, younger and older juvenile, and adult mean jaw shapes.

To reintroduce absolute size data and compare actual change in ramus and corpus lengths and widths with age, linear measurements were made on the same mandibles that had been digitized and radiographed. Time constraints on data collection limited the number of specimens that could be radiographed and landmarked as well as measured. Subsequently, sample sizes varied with the type of analysis (Table 4).

**Figure 3:**
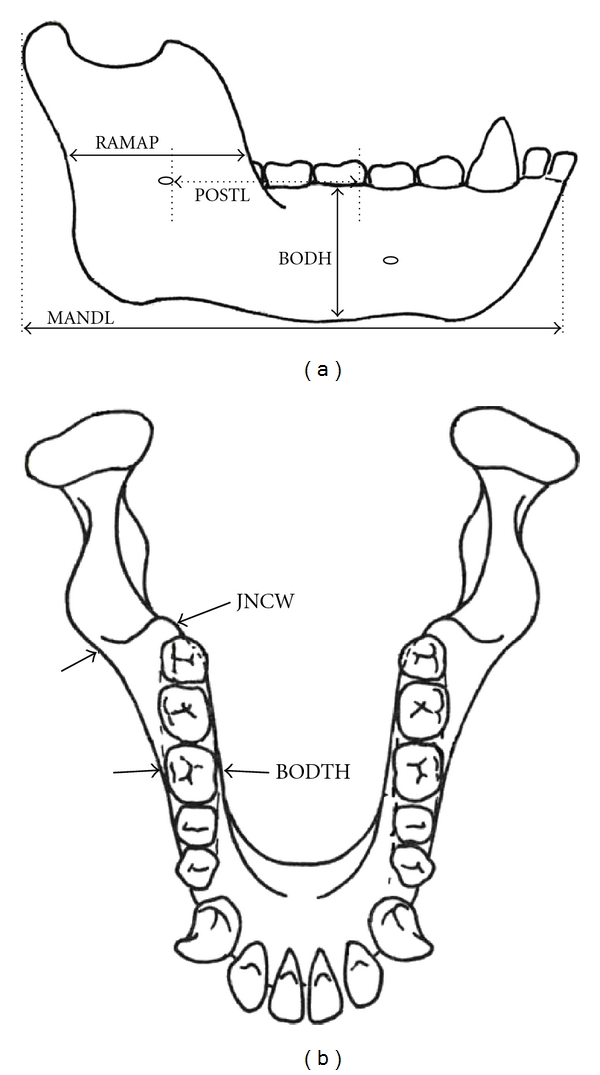
Mandible linear measurements: (a) buccal view and (b) occlusal view.

The mental and mandible foramina were used to subdivide the corpus into proximal and distal parts in order to more precisely study growth rates, particularly in the area of the corpus that housed the permanent molars. The foramina were chosen because they are homologous structures that are immediately recognizable and remain relatively static during growth. Further, the decision to subdivide the mandible near M1 or the mental foramen was informed by other studies showing functional and/or developmental modularity of the lower jaw at this point along the corpus [20, 38–41]. Five measurements (Table 5, Figure 3) were taken from the right side of each mandible unless it was damaged, in which case the left side was used. Measurements were made using manual callipers and an osteometric board and were rounded to the hundredth decimal place. Measurement data were analysed with Microsoft Excel (MS Windows 2003) and SPSS software (IBM version 19).

Tests of linearity (means comparison, ANOVA SPSS v.19) confirmed that the scatters of the bivariate plots of mandible dimensions against age (ARDA) and other jaw measurements were linear, enabling the comparison of slopes and absolute growth rates of the corpus. Also, plots of residuals showed random distributions around zero as would be expected for linear trends. Further, linear regression analysis showed strong goodness of fit for the bivariate plots. Where the data conform less tightly to a linear distribution is for older juveniles of all three species. This may be because stages of tooth mineralization and eruption are most varied and variable during this time period, and thus age estimates are more error prone. To pre-empt skewing due to the age distribution of each data set [42], individuals with no less than fully emerged deciduous dentitions or no more than completely mineralized M_3_ crowns were included (Table 4). An ANCOVA is statistically determined within a 95% confidence interval if growth rates (slopes of age plotted against a given jaw measurement) differed significantly among taxa.

## 4. Results

### 4.1. From Infancy, *Papio* and *Pan* Mandible Shapes and Ontogenetic Trajectories Differ Statistically in Mean Mandible Shape

**Figure 4:**
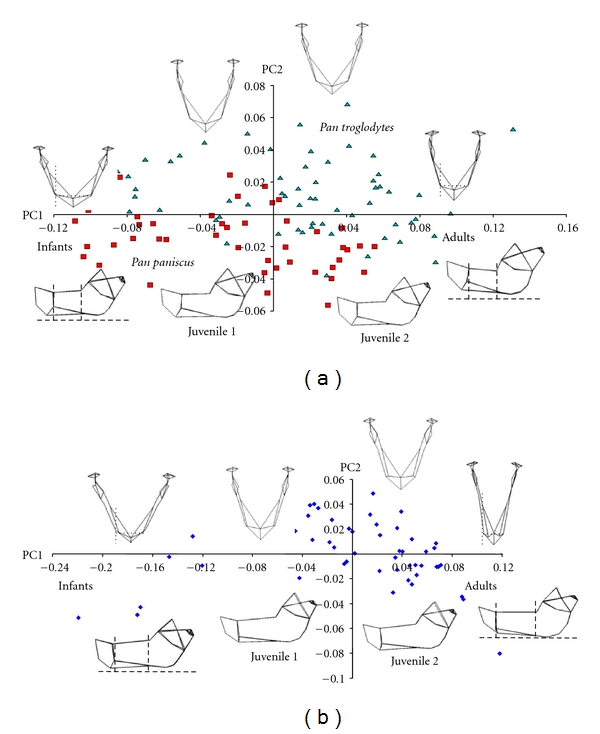
A principal components analysis of mean mandible shape in infant, juvenile, and adult *Pan* (a) and *Papio* (b) across PC1 and PC2 described significant shape differences between genera. Occlusal (upper) and buccal (lower) views of “wire frames” illustrate mean mandible shape for each of the four age groups. Infants are at left and adults are at right. Relative to corpus breadth across the mental foramina, the corpus lengthens much more in *Papio* than in *Pan* (dotted lines). Growth in anteroposterior ramus width and corpus length distal to the mental foramen contributes more to adult jaw proportions in *Pan* than in *Papio*. In *Papio*, corpus growth proximal to the mental foramen contributes relatively more (dashed lines). Red squares, *Pan paniscus*; teal triangles, *Pan troglodytes*; blue diamonds, *Papio anubis*.

After a PCA of all mandible landmarks, permutations tests showed that mean mandible shapes were significantly different between *Papio* and *Pan* from infancy through to adulthood (*P* < .001 for all four age groups). Across principal components (PCs) 1–4, shape variance was not associated with sexual dimorphism in any age group for either genus. Shape differences between genera described by PC5 and higher were very subtle and thus are not reported here. Between *Papio* and *Pan*, the same six major differences in mean mandible shape were noted in each of the four age groups (Figures 4(a) and 4(b)). Note that because absolute specimen size was removed by GPA, the following references to length, height, and width are only relative.

First, as described by PC1, proximal and distal parts of the corpus contributed differently to total corpus length. In *Papio*, a subsection of the corpus (proximal corpus) defined from the mental foramen proximal (backward) to the anterior edge of the ascending ramus was longer than the length of the corpus (distal corpus) distal to (in front of) the mental foramen. The opposite was seen in *Pan*, where the distal corpus was longer relative to the proximal corpus. Related to this, the mental foramen shifted distally with increasing age in *Papio* but was largely static in *Pan*. Second, described by PC2, the mandible condyle was taller than the coronoid process in *Pan,* while the inverse was seen in *Papio*. Also, the buccal face of the corpus was concave in *Papio* but relatively flat in *Pan*. Fourth, anteroposterior (AP) ramus width was smaller relative to corpus length in* Papio* compared to *Pan*. This difference between genera increased with age. Fifth, across PC3, the surface area of the gonial angle was larger in *Papio* than *Pan*. Finally, PC4 described a slightly more acute gonial angle in *Pan*, although the gonial angles of both genera exceeded 90° in infants to adults.

### 4.2. Mandible Shape Trajectory

**Figure 5:**
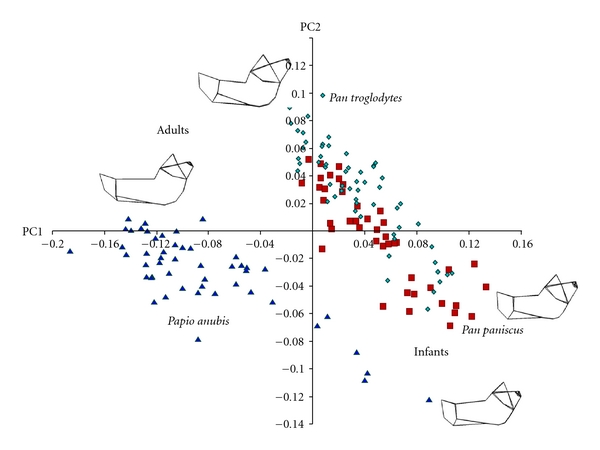
On the plot of mandible shape variance across PCs 1 and 2 for *Papio* and *Pan*, PC1 describes variance that is closely associated with ontogenetic growth. The scatters of *Papio* and *Pan* do not overlap; the angle between both scatters is statistically significant, indicating that the mandibles of each genus follow different ontogenetic trajectories to different adult shapes (wireframes, upper left). Both scatters are linear. Thus, mandible growth trajectory is established at or before birth. Red squares, *Pan paniscus*; teal diamonds, *Pan troglodytes*; blue triangles, *Papio anubis*.

Across PC1, individuals were distributed approximately by their centroid size (Figure 5). Statistical tests confirmed that PC1 and centroid size correlated strongly in *Papio* (0.83) and *Pan* (0.90) (*P* < .0001). Thus, PC1 described shape variance related to mandible ontogeny. Further, PC1 described 49.9% of the total variance in mandible shape between genera. Thus, half of the total shape variance in the lower jaw was directly related to its ontogenetic growth. PC2 also correlated positively with centroid size in *Pan* (0.87) and *Papio* (0.77) (*P* < .0001), but PC2 described only 16.8% of the total shape variance. Subsequent PCs 3–6 described 5.12%, 3.27%, 2.34%, and 2.20% of the remaining total shape variance, respectively. PCs 7 and higher each described less than 2% of the total variance. Shape change described only by PCs 1, 2, and 3 statistically distinguished *Papio* from *Pan*.

Vectors of baboon and ape mandible shape variance across PCs 1 and 2 were statistically different as confirmed by a permutation test (*P* < .0009). Again, as absolute size was removed by a GPA prior to a PCA, changes in jaw proportion are discussed here in relative terms only. These changes are similar to what was seen in the multivariate analysis of mean mandible shape. Across PC1, the mandible lengthened visibly during development in *Pan* and *Papio*, but much more in *Papio*. In the baboon, the extension of the corpus proximal to the mental foramen as far back as the anterior margin of the ramus contributed to a much greater proportion of adult corpus length. In contrast, this subsection of the corpus did not elongate substantially in either the chimpanzee or the bonobo. Rather, in *Pan* corpus length distal to the mental foramen increased notably. The greater length of the adult baboon mandible was due to growth across the entire length of the corpus, in both the distal and proximal subsections of the jaw (Figure 5). Conversely, growth in the distal subsection of the corpus and in anteroposterior ramus width contributed more to final adult mandible length in both apes (Figures 4(a) and 5). In *Pan*, the proximal subsection (from the mental foramen to the ascending ramus) changed relatively little over time (Figures 4(b) and 5).

### 4.3. Growth Rates Differ Regionally within the Corpus and between *Papio* and *Pan*

**Figure 6:**
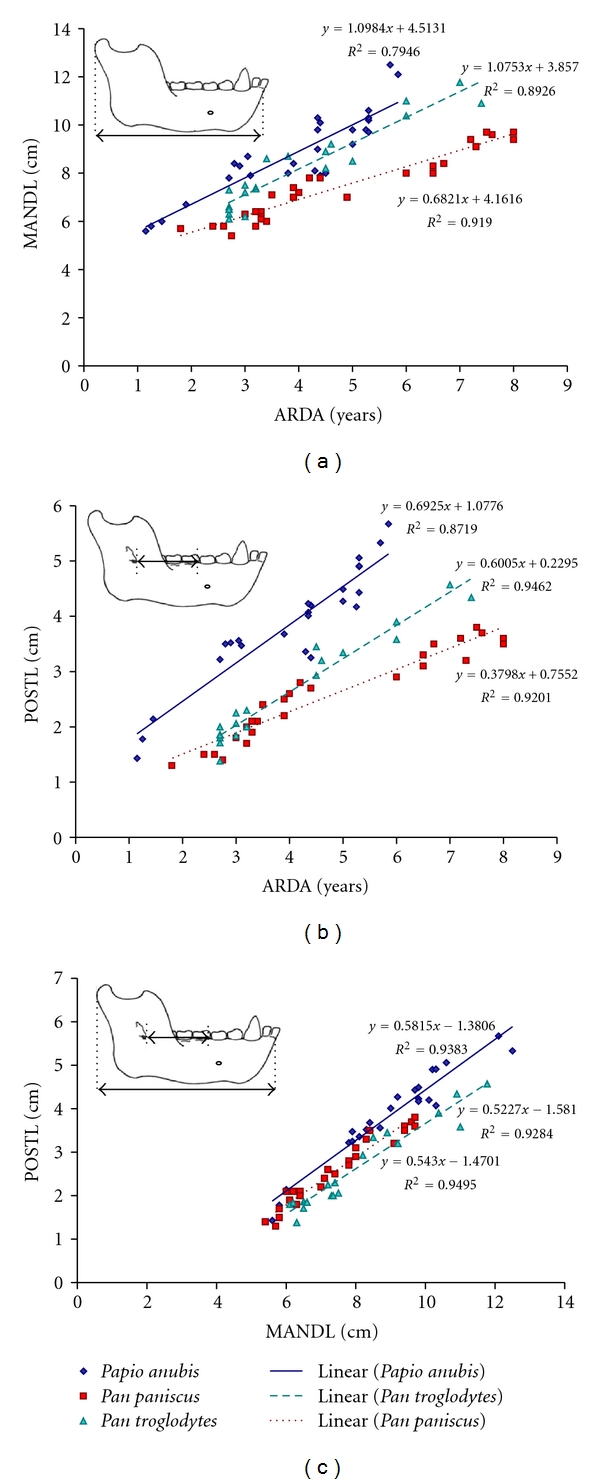
(a) Total mandible length (MANDL) grew at similar rates in *Papio* and *Pan troglodytes*, but more slowly and to shorter adult lengths in *Pan paniscus*. (b) Posterior corpus length (POSTL), where the permanent molars develop, grew to the longest adult lengths at the fastest rates in *Papio*. The inverse was seen in *P*. *paniscus*. (c) In all three species, POSTL grew to about half the length of MANDL. Red squares, *Pan paniscus*; teal triangles, *Pan troglodytes*; blue diamonds, *Papio anubis*.

**Figure 7:**
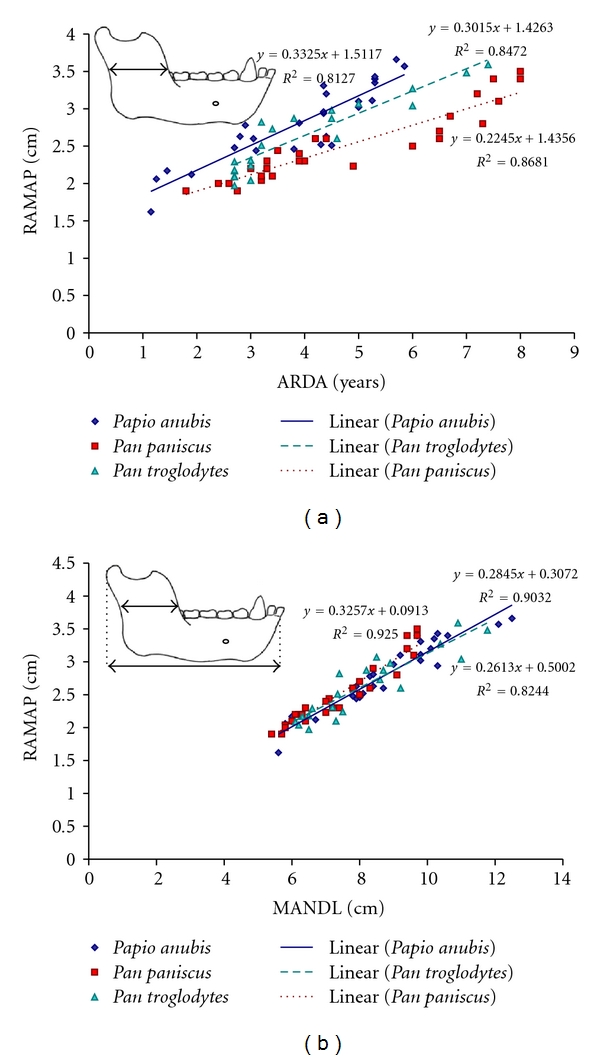
(a) Anteroposterior ramus width (RAMAP) increased fastest with age (ARDA) in *Papio* and *Pan troglodytes*, and more slowly in *Pan paniscus*. (b) RAMAP contributed to around one quarter of adult mandible length in *Papio* and *P. troglodytes* and closer to one-third in *P. paniscus*. Red squares, *Pan paniscus*; teal triangles, *Pan troglodytes*; blue diamonds, *Papio anubis*.

Statistical shape analyses showed significantly different mean mandible shapes and growth trajectories between *Pan* and *Papio*. Next, absolute linear measurements of *Papio* and *Pan* mandibles (Tables 6, 7, and 8, Figures 6-7) were statistically compared to assess their rates of growth from infancy to adulthood. 

In *Papio*, total mandible length (MANDL) increased about 1 cm for every year of postnatal growth (Figure 6(a)). By 6 years of age, MANDL had more than doubled its infant length in the baboon. By the same age, MANDL had grown only an additional 75% of infant dimensions in bonobos and only about 60% in chimpanzees. In infant apes, MANDL was about 1 cm greater in the chimpanzee compared to the bonobo. This size difference approximately doubled between *P. paniscus* and *P. troglodytes* by early adulthood. In a plot of total mandible length against age (ARDA), the slopes (rates of growth) were almost equivalent between *Papio* and *Pan troglodytes *(*m* = 1.1 for both; *R* ^2^ = 0.80 and 0.89, resp.) (Figure 6(a)). MANDL grew only somewhat more slowly in the bonobo (*m* = 0.7, *R* ^2^ = 0.92). Thus, total mandible length grew at about equal rates in *P. troglodytes* and *Papio* and about 2/3 slower in *P. paniscus*. An analysis of covariance (ANCOVA) of MANDL versus ARDA showed statistical differences among species (*F*-value = 65.2, *P* < .001) and between genera (*F*-value = 25.57, *P* < .001).

Next, growth rates for the posterior length of the corpus housing the permanent molars (POSTL), measured from the anterior margin of the M_1_ crypt/tooth back to the mandible foramen (Figure 3), were compared. The slope of POSTL against age (ARDA) was slightly larger (thus faster growth rate) in *Papio* (*m* = 0.7) compared to *Pan* (*P*. *troglodytes, m* = 0.6, *P*. *paniscus, m* = 0.4) (Figure 6(b)). Distributions differed significantly between *Papio* and *Pan* (ANCOVA, *F*-value = 153.35, *P* < .001) and among all three species (*F*-value = 130.8, *P* < .001). As with MANDL, correlation between POSTL and ARDA was strong for bonobos (*R* ^2^ = 0.92), chimpanzees (*R* ^2^ = 0.95), and baboons (*R* ^2^ = 0.87). In infancy, POSTL measured about the same in the apes (less than 0.5 cm longer in chimpanzees versus bonobos) and was half again longer in the baboon. By adulthood, POSTL had almost quadrupled in *Papio*. In contrast, POSTL had, at most, tripled in *Pan* and was almost 1 cm longer in young adult chimpanzees compared to bonobos. The length of this molar-bearing region of the corpus also increased at a slightly faster rate in chimpanzees (*m* = 0.6) versus bonobos (*m* = 0.4) (Figure 6(b)). In adult *Papio*, POSTL was about half again longer compared to adult *Pan*.

Even from infancy, the molar-bearing region of the corpus (POSTL) contributed to a greater proportion of total mandible length (MANDL) in *Papio* compared to *Pan* (Figure 6(c)). By adulthood, POSTL made up about  1/2 of MANDL in *Papio* and just over 1/3 of MANDL in *Pan*. POSTL correlated very strongly with MANDL in all three species (bonobo, *R* ^2^ = 0.95; chimpanzee,  *R* ^2^ = 0.93; baboon, *R* ^2^ = 0.94). In both *Papio* and *Pan*, POSTL grew at about half the rate of MANDL (bonobo, *m* = 0.54; chimpanzee, *m* = 0.52); baboon, *m* = 0.58) although growth rates of POSTL relative to MANDL were statistically different between genera (ANCOVA, *F*-value = 48.17, *P* < .001) and among the three species (*F*-value = 45.2, *P* < .001).

As with total mandible length, corpus height measured at M_1_ (BODH) grew slightly but significantly faster in baboons (*m* = 0.3, *R* ^2^ = 0.74) than in chimpanzees (*m* = 0.2, *R* ^2^ = 0.85) or bonobos (*m* = 0.1, *R* ^2^ = 0.86) (ANCOVA was significant for all species *F* = 67.5, *P* < .001; genera *F* = 45, *P* < .001). BODH was also absolutely tallest in baboons and shortest in bonobos. Across age groups, there was very little change in corpus width measured at M_1_ (BODTH), which increased at most 0.5 cm from infancy to adulthood in all three species with correlations between width and age (*R* ^2^ = 0.24, 0.05, and 0.27 in *Papio*, *P. paniscus,* and *P. troglodytes*, resp.) that were so weak as to be almost meaningless. Variation in growth rate of BODTH was statistically significant among species (ANCOVA, *F*-value = 9.53, *P* < .001) but not between genera (*F*-value = 0.26, *P* = .78). The width of the corpus at its junction with the ascending ramus (JNCW) is an area in which molar crypts and teeth may start to form, likely to compensate for a lack of space in the corpus proper. Rates of widening for JNCW were fastest in *Pan troglodytes* (*m* = 0.1) and slowest in *P. paniscus* (*m* = 0.05) (*Papio m* = 0.07). While correlation with ARDA was strong in chimpanzees (*R* ^2^ = 0.78), it was moderate for bonobos and baboons (*R* ^2^ = 0.55 and 0.49, resp.). There was very little change in JNCW over time and no significant difference between *Papio* and *Pan* (ANCOVA, *F*-value = 0.61, *P* = .55) but a statistical difference among all 3 species (ANCOVA, *F*-value = 7.49, *P* = .001).

Anteroposterior ramus width (RAMAP) increased at similar rates in baboons, chimpanzees (*m* ≈ 0.3, *R* ^2^ = 0.81 and 0.85, resp.), and bonobos (*m* = 0.2, *R* ^2^ = 0.87) (Figure 7(a)). However, covariance was statistically different among all species (*F*-value = 42.5, *P* < .001) and between genera (*F*-value = 21.1, *P* < .001). For all species across all age groups, RAMAP remained very similar in proportion to total mandible length (about 1/3 of MANDL) (Figure 7(b)). Ramus width made up a slightly greater proportion of total mandible length in *Pan* compared to *Papio*. Based on the radiographic data, permanent molar crypts and teeth begin to form in not only the mandible corpus but also the anterior portion of the ascending ramus in both genera.

## 5. Discussion

Mandible shape was statistically distinct between *Papio* and *Pan* from infancy through adulthood. Thus, as might be expected, genus-level differences in mandible shape were established perinatally and probably even before birth. Mandible ontogenetic trajectory was also discrete between *Papio* and *Pan*. As such, genus-level differences in adult mandible morphology arose from a combination of differences in jaw shape near birth that were augmented and entrenched by different postnatal growth patterns. These results contrast with a recent report that while mandible shape differed between sister species *Pan paniscus* and *P. troglodytes* from infancy, postnatal mandible growth pattern did not [43]. Thus, while a single mandible growth pattern may be shared by species within a genus, jaw growth patterns would appear to differ between genera. Earlier work contrasting *Gorilla* and *Pan* mandible morphology and growth supports this idea [20]. While yet to be proven if mandible growth pattern is conserved within a genus, then, data allowing, it would be a useful taxonomic indicator at the genus level. Perhaps more interesting is that this finding implies a decoupling of dental development and jaw development, where dental development schedule and pattern are species specific, but jaw development pattern is not necessarily so. These findings are intriguing and more work needs to be done to both confirm them in other primate taxa and to explore the evolutionary and developmental mechanisms underlying them.

Compared to chimpanzees, baboon molars mineralize within shorter periods of time and become functional at much younger chronological ages [4, 25, 26, 44, 45]. The baboon's permanent molars are also longer anteroposteriorly than those of the apes. For this reason, rates of lower jaw growth were expected to be accelerated in *Papio* in order to accommodate earlier molar development and emergence. This expectation was confirmed here by faster growth in corpus length and height and, to a degree, ramus width, in *Papio* compared to *Pan*. Specifically, the length of the corpus housing the permanent molars (POSTL) contributed to a greater proportion of total jaw length (MANDL) in *Papio* compared to *Pan*. POSTL as well as MANDL also grew at faster rates in *Papio*. Thus, in the baboon faster growth rates of the corpus, particularly the part of the alveolus that houses the permanent molars correlates positively with longer molar crown length and earlier timing of tooth initiation/emergence. Variation in growth rates across the length of the corpus suggests that if the permanent dentition is driving corpus growth [21], then different permanent teeth (i.e., antemolar versus molar) influence the alveolus in different ways.

From infancy to adulthood, there was very little, although still significant, change in corpus width (BODTH, taken at M_1_ or at the site of its dilating gubernaculum) in either genus. The width of the corpus measured at its junction with the ascending ramus (JNCW) also increased minimally with age in *Papio* and *Pan*. Thus, corpus width achieved adult size earlier in life and did not appear to contribute much new space for the permanent molars. Throughout growth and certainly by young adulthood, anteroposterior (A-P) ramus width (RAMAP) contributed to about 1/3 of the total mandible length in the apes and to between 1/3 and 1/4 in the baboon. Based on the radiographic data, permanent molar crypts and initiating teeth formed in the anterior rami of both *Pan* and *Papio*. However, variation in A-P ramus width would seem to be much more an outcome of biomechanical demands than a response to the spatial requirements of the developing molars.

Mandible shape is in large part entrenched at a fundamental, molecular level [46–50], but adult jaw morphology arises from a complex combination of genetic, epigenetic and biomechanical inputs [51]. The functional demands of mastication and a biomechanical balance of stability, manoeuvrability, and force significantly impact mandible form [52–54]. This includes the different vectors of force exerted on the growing mandible by the developing masticatory muscles [55–59]. Broadly, taxonomic differences in gonial angle size, ramus height, and condyle position are related to the mechanical efficiency of the masticatory muscles and the maximum occlusal forces they exert [60–64]. Thus, differences in gonial shape between *Papio* and *Pan* likely reflect the different positions and masses of masticatory muscles that exert different vectors of force on the bone in this gonial region and across the ramus. These effects may be conditional, perhaps on mandible proportion and size [53, 60, 65] and proportion of “slow” and “fast” muscle fibre types [57]. While diets vary compositionally between bonobos and chimpanzees, what they eat is rather the same: both species dine on large amounts of sweet arboreal fruits, followed by lesser quantities of herbs and herbivorous vegetation [66–70]. Typically, baboons predominantly consume vegetable food year round, the bulk of which is made up by grasses [71, 72]. Seasonal fruits and rhizomes make up the second largest proportion of the baboon's diet [71]. However, diet composition can vary according to grassland versus forested habitat [73]. These differences in food type and texture and diet composition inevitably translate into different strengths and forces of masticatory muscle action and varying effects on mandible form.

 Finally, as is the focus of this paper, the growing mandible must accommodate the mineralizing permanent dentition and its emergence into proper functional occlusion. Tooth shape and size account for another part of the taxonomic variation in corpus length and width [60]. African ape edentulous jawbone was more plastic than was alveolar bone, and regional differences in jaw function appear to have translated into developmental decoupling (or vice versa) among these various regions such as mandible corpus, ramus, condyle, and alveolus. However, this report [20] did not mention regional differences in growth rates within the corpus alone. 

There is evidence in humans of variation in postnatal growth rates across the mandible [39] and a primary growth centre near the position of M_1_ [38]. A 3D study of the developing human mandible described the first ossification centre not far from dm1, in the position of the future mental foramen and other small centres inferior to the posterior ends of Meckel's cartilage [40]. Also, the development of Meckel's cartilage initiates in a similar area of the molar tooth bud region [41]. Another study of prenatal human mandibles identified a growth centre at the position of the first deciduous molar germ (dm1) [38] near the mental foramen, from which trabecular bone growth radiates outward—largely, proximally, and distally—across the corpus. A similar configuration was reported in human fetuses [74], where, again, ossification proceeds away from this centre in different directions [75]. Hypothetically, this growth centre might be a mechanism that allows or spurs corpus growth rates to vary on either side of it. Unfortunately, even if such growth centres and radial lines existed in the postnatal ape or baboon mandibles imaged here, then the higher exposure times and voltages necessary to properly capture the developing dental tissues all but obliterated the more delicate details of the trabeculae.

There are several detailed reports of specific local regulation of osteogenesis in the alveolus [76–78] and, more recently, the ramus [79]. For example, in the alveolus, parathyroid hormone-related protein (PTHrP) is critical to promote the osteoclast formation that maintains a bone-free area around each tooth [77]. In the periosteum of the ramus, various genes and their products including BMP2, osteocalcin, alkaline phosphotase, OPG/RANKL, and runx2 help to regionally modulate apposition (osteoblast activity) and resorption (osteoclast activity) [79]. No published study of regional variation in dermal bone growth rate was found to help explain how growth rate might vary across the length of the mandible corpus. Regional variation in postnatal growth rates has been observed in long bones [80, 81] and may be related to localized mechanical stresses [82] and/or variation in growth factor (i.e., IGF-IR) expression in the growth plates [81]. Regional variation in corpus growth rate is not far fetched when one considers that there is regional variation in jawbone density based on loading [83]; both kinds of local change (biomechanical and developmental) in bone growth likely occur via similar osteogenic mechanisms. Variation in osteoblast and osteoclast activity across the jawbone may drive differences in corpus elongation rates in response to signalling or hormone gradients. Faster lengthening would be expected to correlate with higher numbers of proliferating osteocytes, among other factors.

The idea that one or more growth centres in the mandible facilitate different growth rates across the corpus has interesting implications for ontogenetic and evolutionary changes in jaw form in response to altered tooth proportion and/or number. Selection seems to act more strongly on the teeth than the mandible, where the face responds to change in the dentition rather than vice versa [17–19]. Segregated growth centres could facilitate faster and/or more precise local changes in mandible growth to accommodate specific odontological modifications while minimizing disruptions to adjacent, unimplicated regions of the jaw. While this is an interesting idea, much work needs to be done to test it.

## 6. Conclusions

Between *Pan* and *Papio*, mandible shape and ontogeny are statistically distinct. Thus, genus-level differences in mandible morphology are not only present from birth but also arise and become entrenched via different postnatal growth trajectories. The mandible corpus lengthens at different rates between genera. Further, growth rate varies locally within the corpus. Regional variation in corpus growth rate and innate differences in corpus proportions appear to act in tandem to create space in a timely way for the permanent molars in *Papio* and *Pan*. Local growth rates across the corpus may evolve alongside changes in molar morphology and developmental timing to help the teeth and jaw remain ontogenetically and functionally compatible.

## Figures and Tables

**Table 1:** Age groups and sexes of the specimens belonging to the three primate taxa included in this study. I: infant; J1: juvenile 1; J2: juvenile 2; A: adult.

Taxon	*Papio anubis*				*Pan paniscus*				*Pan troglodytes*			
Age group	I	J1	J2	A	I	J1	J2	A	I	J1	J2	A
*Sex*												
Male	3	6	10	6	7	8	6	1	6	12	3	4
Female	3	7	4	9	5	4	4	4	9	6	3	4
Unknown	1	1	1	0	3	1	1	0	7	5	0	0

Total	7	14	15	15	15	13	11	5	22	23	6	8

**Table 2:** Age groups based on dental development for *Papio* and *Pan*.

	Age groups (in years)			
Taxon	Infant	Juvenile 1	Juvenile 2	Adult
*Papio*	0–2.0	2.01–4.5	4.51–7.0	+7.0
*Pan*	0–3.5	3.51–7.0	7.01–10.5	+10.5

**Table 3:** Mandible landmarks.

Number: right, left	Description
1, 21	Superolateral-most tip of the coronoid process
2, 22	Inferior-most point of the mandible notch
3, 23	Anterior-most tip of the condyle
4, 24	Lateral-most tip of the condyle
5, 25	Posterior-most tip of the condyle
6, 26	Medial-most tip of the condyle
7, 27	Superior-most tip on the articular surface of the condyle
8, 28	Deepest concavity of the posterior border of the ascending ramus
9, 29	Point opposite landmark 8/28, on the anterior border of the ascending ramus
10, 30	Apex of lingula, or, if lingula is undefinable or absent, the anterosuperior-most margin of the mandible foramen
11, 31	Point on the posterior border of the ramus just superior to the blending of the ramus into the gonial angle or the point at which a tangent leaves the posterior border of the ramus
12, 32	Posteriorly, the point on the gonial angle that is the apex of the (90°) angle formed by landmarks 11/31 and 13/33
13, 33	Inferiorly, the point along the gonial angle where a tangent leaves the inferior margin of the mandible
14, 34	Superiorly directed indentation of the inferior border of the mandible corpus, just anterior to landmark 13/33
15, 35	Point at which the ascending ramus meets and obscures the corpus, in lateral view along the alveolar bone
16, 36	Mental foramen, midpoint at the level of the surface of the mandible corpus
17, 37	Point on the alveolar border of the mandible corpus directly superior to landmark 16/36
18	Midpoint between the central incisors at the superior-most tip of the alveolar bone
19	Anterior-most projection of the subalveolar bone in the mental region along the midline
20	Symphyseal midpoint of the inferior margin of the mandible corpus, directly inferior to the areas of attachment of the geniohyoid and genioglossus muscles

**Table 4:** Specimens per taxon included in the multivariate and bivariate analyses.

Analysis	Number of individuals per taxon included		
	*Papio anubis*	*Pan troglodytes*	*Pan paniscus*
Aging (ARDA)	52	59	44
3D Multivariate mandible shape	50	56	44
3D Multivariate mandible shape change	52	58	44
2D Bivariate mandible dimensions	27	21	28

**Table 5:** Mandible measurements.

Measurement (abbreviation)	Description
Body width (BODTH)	The minimum thickness of the corpus measured across M_1_ or the gubernaculum of the same tooth with the jaws of the callipers orthogonal to the occlusal plane
Body height (BODH)	Corpus height from the inferior-most point of the crest of the buccal alveolar bone opposite the mesiobuccal root of M_1_, or the inferior border of M_1_ gubernaculum to the lower border of the mandible
Mandible length (MANDL)	The minimum anteroposterior length of the mandible measured between a line perpendicular to the posterior-most points of the condyles to a line perpendicular to the anterior-most point of the symphysis (measured with a mandible board)
Posterior length (POSTL)	The length of the mandible from the anterior inferior margin of the mandible foramen to the distal margin of dm_2_ (infant or juvenile) or from the same margin to the anterior margin of M_1_ (once dm_2_ is shed) measured on the lingual side
Junction width (JNCW)	The maximum width of the corpus at the junction of the ramus and the corpus with the caliper jaw orthogonal to the occlusal plane
Ramus width (RAMAP)	The minimum anteroposterior width of the ascending ramus

**Table 6:** Raw mandible measurement data for *Papio anubis*. I: infant; J1: juvenile 1; J2: juvenile 2.

Specimen ID	ARDA (years)	Age group	Mandible measurements (cm)					
			BODTH	BODH	RAMAP	MANDL	POSTL	JNCW
1953.655/32	1.2	I	0.98	1.20	1.62	5.60	1.43	1.04
92.21/76	1.5	I	1.10	1.34	2.17	6.00	2.14	1.00
92.182/75	1.3	I	0.97	1.34	2.06	5.80	1.78	0.81
92.22/79	1.9	I	1.06	1.44	2.12	6.70	1.35	1.11
1930.3.4.1/57	2.7	J1	1.06	1.71	2.48	7.80	3.22	1.16
1937.7.24.1/59	2.8	J1	1.24	1.87	2.63	8.40	3.50	1.35
1914.3.8.1/42	3.1	J1	1.10	1.85	2.60	8.70	3.56	1.18
1967.1152/40	3.1	J1	1.09	1.84	2.44	7.90	3.47	1.18
92.3/74	2.9	J1	1.09	2.00	2.78	8.30	3.52	1.21
92.31/81	3.9	J1	1.07	2.05	2.81	8.40	3.68	1.07
92.14/80	3.8	J1	1.15	1.78	2.46	8.00	2.47	0.70
1939.1034/51	4.4	J1	1.18	2.40	3.20	10.10	4.20	1.15
1855.12.26.32/45	4.4	J1	1.12	2.10	2.94	10.30	4.07	1.33
1931.4.1.2/64	4.4	J1	1.16	2.30	3.31	9.80	4.23	1.30
92.141/72	4.4	J1	1.09	2.28	2.96	9.00	4.01	1.17
1967.1151/41	4.3	J1	0.98	1.70	2.52	8.10	3.36	1.03
92.24/77	4.4	J1	1.03	1.67	2.63	7.90	3.25	1.20
92.23/73	4.5	J1	1.05	1.86	2.51	8.00	2.57	1.10
92.15/71	5.0	J2	1.15	2.14	3.10	9.20	4.27	1.14
1913.10.18.1/47	5.3	J2	1.08	2.52	3.35	10.20	4.90	1.23
92.36/78	5.0	J2	1.08	2.23	3.02	9.80	4.49	1.21
92.12/70	5.3	J2	1.12	2.75	3.40	10.60	5.06	1.42
1924.8.6.14/43	5.3	J2	1.22	2.23	3.11	9.80	4.17	1.39
1939.55/44	5.3	J2	1.22	2.20	3.40	9.70	4.43	1.36
1928.6.3.1/35	5.3	J2	1.11	2.34	3.43	10.30	4.91	1.43
1939.3451/67	5.7	J2	1.22	3.09	3.66	12.50	5.33	1.36
1973.18.12/65	5.9	J2	1.14	2.78	3.57	12.10	5.67	1.35

**Table 7:** Raw mandible measurement data for *pan trolodytes*. I: infant; J1: juvenile 1; J2: juvenile 2.

Specimen ID	ARDA (years)	Age groups	Mandible measurements (cm)					
			BODTH	BODH	RAMAP	MANDL	POSTL	JNCW
1939.1000/15	2.7	I	1.04	1.44	2.17	6.30	1.38	0.95
1948.439/9	2.7	I	1.13	1.31	1.97	6.50	1.86	1.06
1939.1004/17	2.7	I	1.22	1.40	2.10	7.30	2.00	1.18
1986.221/6	2.7	I	1.05	1.14	2.09	6.10	1.80	1.10
1948.438/11	2.7	I	0.96	1.31	2.29	6.60	1.85	1.16
1986.217/100	2.7	I	1.24	1.39	2.18	6.50	1.71	1.17
1939.915[b]/10	3	I	1.06	1.21	2.04	6.20	1.84	1.01
1939.979/8	3	I	1.22	1.47	2.24	7.50	2.06	1.06
1939.997/18	3.2	I	1.20	1.75	2.82	7.40	2.30	1.20
1980.341/5	3	I	1.24	1.52	2.31	7.20	2.25	1.16
1939.1003/16	3.2	I	1.12	1.65	2.51	7.35	2.00	1.20
1939.3373/19	3.4	I	1.43	1.69	2.73	8.60	3.26	1.45
1846.10.23.11/7	3.8	J1	1.23	1.57	2.87	8.70	3.32	1.29
1939.1002/4	4.5	J1	1.20	1.79	2.87	8.20	2.93	1.15
1980.339/3	4.6	J1	1.24	1.71	2.60	9.21	3.20	1.08
1939.998/20	4.5	J1	1.37	1.94	2.98	8.90	3.45	1.38
1926.11.18.1/24	6	J1	1.26	2.08	3.27	10.38	3.90	1.39
1901.8.9.9/25	6	J1	1.31	1.94	3.04	11.00	3.58	1.47
1887.12.1.3/12	7.4	J2	1.25	2.27	3.59	10.90	4.34	1.71
1989.326/27	7	J1	1.20	2.52	3.48	11.77	4.57	1.55
1989.327/23	5	J1	1.23	1.85	3.07	8.50	3.34	1.33

**Table 8:** Raw mandible measurement data for *Pan paniscus*. I: infant; J1: juvenile 1; J2: juvenile 2.

Specimen ID	ARDA (years)	Age group	Mandible measurements (cm)					
			BODTH	BODH	RAMAP	MANDL	POSTL	JNCW
18050/6	3.2	I	1.00	1.30	2.04	5.80	1.70	1.00
11293/8	1.8	I	1.02	1.20	1.90	5.70	1.30	1.05
12087/9	3.3	I	1.10	1.50	2.30	6.40	2.10	1.13
22336/17	3.4	I	1.15	1.60	2.10	6.00	2.10	1.10
23464/18	3.3	I	1.10	1.30	2.20	6.20	2.10	1.00
26959/25	3.2	I	1.20	1.33	2.10	6.40	2.00	1.10
26958/24	3.3	I	1.10	1.40	2.20	6.10	1.90	1.14
26972/34	2.4	I	0.93	1.10	2.00	5.80	1.50	0.90
26975/36	3.5	I	1.20	1.50	2.44	7.10	2.40	1.30
26976/37	2.6	I	1.10	1.30	2.00	5.80	1.50	1.00
26977/23	3.0	I	1.10	1.40	2.20	6.30	1.80	1.00
26990/0	2.8	I	1.00	1.20	1.90	5.40	1.40	0.93
11528/10	6.0	J1	1.00	1.80	2.50	8.00	2.90	1.30
9369/11	4.9	J1	1.00	1.50	2.23	7.00	1.92	1.10
26936/19	4.0	J1	1.10	1.40	2.30	7.20	2.60	1.10
22908/20	6.5	J1	0.90	1.60	2.60	8.30	3.30	1.10
26968/26	3.9	J1	0.23	1.50	2.40	7.00	2.20	1.30
26970/27	4.2	J1	1.10	1.40	2.60	7.80	2.80	1.20
26982/33	4.4	J1	1.10	1.30	2.60	7.80	2.70	1.20
26969/35	3.9	J1	1.01	1.40	2.30	7.40	2.50	1.10
26988/28	6.5	J1	1.10	1.70	2.70	8.00	3.10	1.20
27001/3	6.7	J1	1.10	1.70	2.90	8.40	3.50	1.20
5374/13	7.3	J2	1.16	1.90	2.80	9.10	3.20	1.26
26947/29	7.6	J2	1.10	1.80	3.10	9.60	3.70	1.30
26971/32	7.5	J2	1.20	1.90	3.40	9.70	3.80	1.40
26994/4	7.2	J2	1.20	1.90	3.20	9.40	3.60	1.40
26993/5	8.0	J2	1.10	1.90	3.40	9.40	3.50	1.20
26996/2	8.0	J2	1.10	2.00	3.50	9.70	3.60	1.34
